# Phenotypic Traits and Probiotic Functions of *Lactiplantibacillus plantarum* Y42 in Planktonic and Biofilm Forms

**DOI:** 10.3390/foods12071516

**Published:** 2023-04-03

**Authors:** Jiayi Li, Guangqing Mu, Yanfeng Tuo

**Affiliations:** 1 School of Food Science and Technology, Dalian Polytechnic University, Dalian 116034, Chinaguangqingmu@163.com (G.M.); 2 Dalian Probiotics Function Research Key Laboratory, Dalian Polytechnic University, Dalian 116034, China

**Keywords:** *Lactiplantibacillus plantarum* Y42, planktonic, biofilm, phenotypic traits, probiotic functions

## Abstract

Bacteria in planktonic and biofilm forms exhibit different phenotypic properties. In this study, the phenotypic traits and probiotic functions of *Lactiplantibacillus plantarum* Y42 in planktonic and biofilm forms were assessed. After 36 h of static culture, scanning electron microscopy and confocal laser scanning microscopy showed that the *L*. *plantarum* Y42 bacterial cells contained interconnected adhesive matter on the surface, forming a ~18 μm layer of dense biofilms. The surface properties of *L*. *plantarum* Y42 in biofilm form, including autoaggregation ability, hydrophobicity, acid-base charge, and adhesiveness, were all higher than those in the planktonic form. Biofilm *L*. *plantarum* Y42 showed a higher tolerance to adverse environmental conditions and a higher survival rate, enzymatic activity, and integrity after vacuum lyophilization. And biofilm *L*. *plantarum* Y42 had higher adhesion to human enterocyte HT-29 cell monolayers, inhibited the expressions of proinflammatory factors IL-6 and TNF-α, and promoted the expressions of the anti-inflammatory factor IL-10 and barrier proteins Claudin-1 and Occludin. In addition, *L*. *plantarum* Y42 in biofilm form can inhibit the adhesion and invasion of *Listeria monocytogenes* ATCC 19115 to HT-29 cell monolayers and is more effective in relieving the inflammatory reactions and injuries of HT-29 cells caused by *L. monocytogenes* ATCC 19115. In conclusion, *L*. *plantarum* Y42 in biofilm form exhibited better probiotic functions compared to that in planktonic form. This indicated that *L. plantarum* Y42 can form biofilms to enhance its probiotic functions, which provided a theoretical basis for better development and utilization of *L. plantarum* Y42.

## 1. Introduction

Bacteria can exist in two forms: planktonic and biofilm [1]. The planktonic state is a form in which bacteria cells can be fully exposed to nutrients for growth and metabolism [2]. The bacteria in biofilm form can irreversibly adhere to the surface of either organic or inorganic material without the help of external forces, propagate, differentiate, and secrete some extracellular polysaccharides, proteins, extracellular DNA, and other polymer matrices, which can wrap the bacteria cells to form organized bacterial groups, which is the main existing form of bacteria in nature [2]. The formation and development of bacterial biofilms are affected by many factors, including strains, surface properties, pH, nutrients, temperature, and adhesive materials [3,4]. The existence of biofilms can not only act as a barrier to create a stable internal environment for the life activities of bacteria and mediate the connection of cell-cell and cell-substrate but also play roles in matter transfer, information transmembrane transfer, and energy conversion [1]. It was reported that the bacteria in the biofilm form behave differently from planktonic bacteria, and the formation of biofilms can promote bacteria’s ability to adapt and be tolerant to adverse environmental conditions [5]. Most studies on biofilms were focused on the pathogenic mechanisms, the elimination of pathogens, and the inhibition of pathogenic biofilm formation [6], while the research on beneficial microbial biofilms was relatively scarce and insufficiently deep [7]. 

The Food and Agriculture Organization/World Health Organization (FAO/WHO) in 2001 defined a probiotic as a type of living microbe, whose adequate intake will generate beneficial effects on the host’s health [8]. The use of probiotics and their effects on human health have been the focus of research as a potential substitute therapy to prevent or relieve diseases caused by pathogenic bacteria [5]. Reportedly, probiotics can inhibit pathogenic bacteria from adhering to the intestinal epithelial cells, regulate intestinal immune functions, improve intestinal barrier functions, and maintain the balance of intestinal flora. The probiotic function was associated with the interaction between probiotics and host enterocytes, and the key lies in the adhesion of probiotics to the enterocytes [9,10,11]. The acid stomach environment, the interaction with bile acid, pressure from host cells, and competition with symbiotic bacteria and pathogens will all make probiotics ineffective rapidly, resulting in a loss of ability to adhere and colonize the intestinal tract [12]. The formed biofilms will make the probiotics superior in terms of survival, colonization, and inhibition effects on pathogens in vivo. Biofilm-state probiotics have attracted much attention as an emerging strategy for the full application of probiotics [13]. 

Previously, research from our team showed the supernatants and coarse proteins of *Lactiplantibacillus plantarum* Y42 culture had inhibitory effects on the biofilm formation and adhesion to HT-29 cell monolayers of *Listeria monocytogenes* ATCC 19115, and further study found that *L*. *plantarum* Y42 can form biofilms [14,15]. Nevertheless, it is not clear whether planktonic and biofilm *L*. *plantarum* Y42 differ in phenotypic traits and probiotic functions. In this study, phenotypic traits and probiotic functions of planktonic and biofilm *L*. *plantarum* Y42 were compared, including the strain morphology, surface properties, lyophilized survival rate, stress tolerance, adhesion ability to HT-29 cell monolayers, and their effects on intestinal barrier function and *L. monocytogenes* ATCC 19115 activity, in order to find the best way for the application of *L*. *plantarum* Y42 as a probiotic. 

## 2. Materials and Methods

### 2.1. Strain and HT-29 Cell

*L. plantarum* Y42 is isolated from Dalian traditional dried fish products. *L. plantarum* Y42 and *L. monocytogenes* ATCC 19115 were both stored in the Dalian Probiotic Function Research Key Laboratory. *L. plantarum* Y42 was inoculated into the de Man-Rogosa-Sharpe (MRS) medium (Land Bridge, China) (1: 100, *v*/*v*) at 37 °C for 36 h. *L. monocytogenes* ATCC 19115 was inoculated into trypticase soy broth (TSB, Land Bridge, China) (1: 100, *v*/*v*) at 37 °C for 24 h. 

Human enterocyte HT-29 cells were stored in the Dalian Probiotic Function Research Key Laboratory. HT-29 cells were grown in RPMI 1640 medium (Meilunbio, Dalian, China) supplemented with 1% penicillin and streptomycin (Meilunbio, Dalian, China), 10% heat-inactivated (56 °C, 30 min) fetal bovine serum (FBS; Meilunbio, Dalian, China), and cultured at 37 °C for 2–4 d in a humidified atmosphere containing 5% CO_2_ and 95% air. HT-29 cells were stained with 0.2% trypan blue and counted using the hemocytometer.

### 2.2. Preparation of Planktonic and Biofilm Strains

The planktonic *L. plantarum* Y42 was obtained by centrifugation after 36 h of shaking incubation at 200 r/min and 37 °C. The preparation of the biofilm formation of *L. plantarum* Y42 was performed on polystyrene microplates. The MRS medium, inoculated with *L. plantarum* Y42, was transferred to a 6-well microtiter plate at 2 mL per well and incubated at 37 °C for 36 h. The final concentrations of both strain types reached 10^10^ CFU/mL. After incubation, the cultures of *L. plantarum* Y42 in planktonic form were collected and centrifugated (8000✕ *g*, 10 min, 4 °C; CR21N, HITACHI, Ibaraki, Japan). For the biofilm *L. plantarum* Y42, the supernatant was discarded, and the wells were gently washed three times with aseptic phosphate buffered saline (PBS, pH 7.3). The biofilm cells were resuspended in a small amount of PBS (pH 7.3) and scraped off with an aseptic scraper, then collected and centrifugated (8000× *g*, 10 min, 4 °C). In this experiment, viable bacteria were counted using the plate counting method. 

### 2.3. Growth Curves and Biofilm Formation of L. plantarum Y42

The planktonic and biofilm *L*. *plantarum* Y42 were cultured on 96-well polystyrene microplates (Sangon Biotech, Shanghai, China), according to the method described in the Section “Preparation of planktonic and biofilm strains.” The OD_600 nm_ values of two forms were determined using an FP-1100-C full-auto growth curve analyzer (Thermo, Vantaa, Finland) every 2 h, and the viable bacteria of *L*. *plantarum* Y42 were counted every 12 h. The biofilm biomass was quantified every 4 h by a crystal violet assay. Briefly, the supernatant from each well was decanted, and the biofilm was washed twice with aseptic PBS (pH 7.3). Then, the biofilms were fixed with 95% (*v*/*v*) anhydrous methanol for 15 min, stained with 2% (*m*/*v*) crystal violet for 5 min, and the cell-bound dye was dissolved with 33% (*v*/*v*) glacial acetic acid, and the OD_595 nm_ values were measured after 30 min.

### 2.4. Factors Affecting the Formation of L. plantarum Y42 Biofilms

In order to investigate the effects of different pHs (6.5, 5.5, 5, 4.5, and 4) in the medium, we applied different sugar additions (100% glucose, 50% glucose + 50% sucrose, lactose, maltose, and fructose, respectively) and culture temperatures (25, 30, 37, 42, and 50 °C). The *L*. *plantarum* Y42 was incubated for 36 h under the above-mentioned conditions, and the OD_595 nm_ was measured using the crystal violet assay.

### 2.5. Morphological Observations of Planktonic and Biofilm L. plantarum Y42

#### 2.5.1. Field-Emission Scanning Electron Microscopy (SEM) 

Two forms of *L*. *plantarum* Y42 were prepared according to the method described in the Section “Preparation of planktonic and biofilm strains.” The cells were resuspended in aseptic PBS (pH 7.3) to a final concentration of 2 × 10^9^ CFU/mL (OD_600nm_ ≈ 1). After that, the cell suspensions were uniformly spread onto aseptic glass cover slides and dried naturally, then fixed with 2.5% glutaraldehyde overnight, rinsed with a 0.1 M sodium acetate buffer solution, rinsed again with distilled water, and dehydrated with an ethanol gradient (20%, 50%, 70%, 90%, 100%) for 5 min each time. After air drying at room temperature, the plates were metal-sprayed, and the cell morphology was observed by field-emission scanning electron microscopy (SEM; JSM-7800F, JEOL, Tokyo, Japan) at magnifications of 10,000× and 30,000×. 

#### 2.5.2. Confocal Laser Scanning Microscopy (CLSM) 

The biofilm specimens of *L. plantarum* Y42 were grown on sterile glass slides at the bottom of 6-well microtiter plates for 36 h. The supernatant was decanted, and the biofilm was washed twice with aseptic PBS (pH 7.3). After drying naturally, the biofilm was stained with the LIVE/DEAD BacLight bacterial viability kit according to the manual. Briefly, appropriate amounts of fluorescent dyes, propidium iodide (PI; diluted 1:8000 with PBS), and SYTO 9 (diluted 1:8000 with PBS), were added to the biofilm at the same time to stain for 30 min in the dark. Then the biofilm was washed with aseptic PBS (pH 7.3) and placed on absorbent paper in the dark until dry. The slide was sealed with fluorescent sealing agents, and the images were collected with confocal laser scanning microscopy (CLSM; LSM880, Carl Zeiss, Oberkochen, Germany) under a 40 mm oil immersion objective. The 3D structure of the biofilm was scanned at 0.4 μm in the z-direction between each x-y image. 

### 2.6. Surface Properties of Planktonic and Biofilm L. plantarum Y42

The planktonic and biofilm *L*. *plantarum* Y42 was resuspended in aseptic PBS (pH 7.3) to a final concentration of 2 × 10^9^ CFU/mL (OD_600nm_ ≈ 1). Auto-aggregation ability and hydrophobicity were measured according to the method of Krausova et al. [16]. And surface charge was measured according to the method described by Yao et al. [17]. In addition, aseptic glass slides containing the planktonic or biofilm *L. plantarum* Y42 were prepared following the method described in Section “Field-emission scanning electron microscopy (SEM)” and fixed in 2.5% glutaraldehyde overnight. The surface adhesiveness of *L*. *plantarum* Y42 in two forms was detected by using an SPM-9700 atomic force microscope (AFM; Shimadzu, Kyoto, Japan) with a force constant of 0.11 N/m. The images and force curves in the constant force mode were collected at a size of 256 × 256 pixels and a scanning speed of 1 Hz. 

### 2.7. Measurement of Environmental Stress Tolerance

To determine the tolerance of two forms of *L*. *plantarum* Y42 to harsh environments, we measured the survival rate of *L*. *plantarum* Y42 treated with different pH, bile salt, and temperatures. We added two forms of *L*. *plantarum* Y42 into sterile MRS medium with varying levels of pH (2, 3, or 4), bile salt concentration (0.5 mg/mL, 1 mg/mL, or 2 mg/mL), and temperature (60 °C, 70 °C, or 80 °C) and determined the viable bacteria by using the viable plate count method after 3 h, with the survival rate calculated as follows: survival cell number/initial cell number × 100%.

### 2.8. Tolerance of Gastro-Intestinal Fluid 

The simulated gastric fluid (SGF) and intestinal fluid (SIF) were prepared according to the method described by Zhang et al. [18]. The planktonic and biofilm *L*. *plantarum* Y42 were cultured as described above and resuspended in PBS (pH 7.3) to a final concentration of 2 × 10^9^ CFU/mL (OD_600nm_ ≈ 1), then collected by centrifugation (8000× *g*, 10 min, 4 °C). Two forms of *L*. *plantarum* Y42 were added to equal amounts of the simulated artificial gastric fluid (SGF) and the simulated intestinal fluid (SIF), and treated for 3 h and 8 h at 37 °C, respectively. After doing a gradient dilution, three suitable dilutions were chosen. The viable plate count method was used to identify live bacteria. The survival rate was calculated as N_1_/N_0_ × 100%, where N1 represents the number of colonies that had formed following the addition of simulated gastric fluid or simulated intestinal fluid (CFU/mL) and N_0_ represents the original number of colonies.

### 2.9. Effects of Vacuum Lyophilization on the Planktonic and Biofilm L. plantarum Y42

The viable bacteria of *L*. *plantarum* Y42 in the planktonic and biofilm forms after vacuum lyophilization were determined according to Wang et al. [19]. The planktonic or biofilm *L*. *plantarum* Y42 was mixed with 3% skim milk as the protective agent, pre-frozen at −80 °C for 12 h, and then lyophilized (cold trap temperature at −60 °C, vacuum degree 5 Pa). The lyophilized bacterial powder was rehydrated with 0.9% NaCl to the volume prior to lyophilization, and the quantity of viable cells was calculated after gradient dilution. The lyophilized survival rate (%) was calculated as: viable bacteria after lyophilization/viable bacteria before lyophilization × 100%. And the enzymatic activity (U) of the planktonic and biofilm *L*. *plantarum* Y42 before and after lyophilization was measured using a lactate dehydrogenase (LDH) kit and a Na^+^ K^+^-ATPase kit (Solarbio, Beijing, China). Simultaneously, the lyophilized bacterial powder of planktonic and biofilm *L. plantarum* Y42 was rehydrated and centrifuged, then the cells were collected, fixed, decolorized, embedded, sectioned, and observed by using a JEM-1200EX transmission electron microscope (TEM; Shimadzu, Kyoto, Japan) [20].

### 2.10. Adhesion of Planktonic and Biofilm L. plantarum Y42 on Human Enterocyte HT-29

#### 2.10.1. Adhesion Ability

According to the approach of Greppi et al. [21], with some modification, the ability of planktonic and biofilm *L*. *plantarum* Y42 to adhere to the HT-29 cell monolayers was evaluated. The final concentration of 2.0 × 10^9^ CFU/mL was achieved by resuspending two forms of *L*. *plantarum* Y42 in RPMI 1640 medium (without antibiotics or fetal bovine serum). The 12-well polystyrene microtiter plate was filled with 2 × 10^5^ cells/mL HT-29 cells, and the cells were then cultured at 37 °C in a humidified environment with 5% CO_2_ and 95% air until a cell monolayer had developed. The cells were washed three times with PBS (pH 7.3) after the broth was removed, and 100 μL planktonic or biofilm *L*. *plantarum* Y42 was added to the wells, respectively. The RPMI 1640 medium was refilled to 1 mL and incubated for 2 h. The broth was discarded, and the cells were washed again with PBS (pH 7.3), 500 μL 0.5% TritonX-100 was added, and the cells were evenly blown to lysate. The solution was transferred to 4.5 mL of normal saline for gradient dilution. The quantity of *L*. *plantarum* Y42 adhering to HT-29 cells was determined by the pouring plate method.

#### 2.10.2. Secretion of Inflammatory Factors

The expression levels of cytokines related to inflammation in HT-29 cells treated with two forms of *L. plantarum* Y42 were measured by using real-time quantitative PCR (RT-qPCR) [22]. Total RNA of the treated HT-29 cells was extracted using an RNA extraction kit (Vazyme, Nanjing, China), and RNA integrity and concentration were assessed using 1.2% agarose gel electrophoresis and a NanoDrop^®^ ND-1000 spectrophotometer (Thermo Scientific, Waltham, MA, USA). The first cDNA strand was synthesized by reverse transcription using HiScript^®^ II Q RT SuperMix for qPCR (+gDNA wiper) kit (Vazyme, Nanjing, China) based on the directions provided by the maker. Then the ChamQ Universal SYBR qPCR Master Mix Kit (Vazyme, Nanjing, China) was used for amplification with the following procedure: 95 °C, 30 s, one cycle; 95 °C, 10 s, 60 °C, 30 s, forty cycles; 95 °C, 15 s, 60 °C, 60 s, 95 °C, 15 s, one cycle. Finally, the specificity of amplification was confirmed by the melting curve. The Ct values of inflammatory factors (IL-6, IL-8, TNF-α, and IL-10) were measured with GAPDH as the housekeeper gene, and the relative expression of each gene was computed according to the 2^−ΔΔCt^ method. Table 1 contains a list of the primer sequences that were investigated. 

#### 2.10.3. Expressions of Tight Junction Proteins

The expression levels of three tight junction proteins (Cloudin-1, Occludin, and ZO-1) in HT-29 cells treated by planktonic and biofilm *L. plantarum* Y42 were measured by RT-qPCR and Western blot (WB). The RT-qPCR was described in the Section “Secretion of inflammatory factors”, while β-actin was used as the housekeeping gene. The WB was conducted according to Gao et al. [23] with certain modifications as follows: HT-29 cells treated with either planktonic or biofilm *L*. *plantarum* Y42 for 2 h were rinsed twice with ice-cold PBS (pH 7.3). Then the lysis buffer solution (RIPA:PMSF = 99:1) was added to the ultrasonic for 15 min. The supernatants were collected after centrifugation (10,000 r, 20 min, 4 °C), and the protein concentrations were measured by using a BCA protein assay kit (Solarbio, Beijing, China) in accordance with the manufacturer’s instructions. A certain volume 2× buffer was added, followed by denaturalization in a boiled water bath for 5 min. Then the solutions were separated using an AE8135 sodium dodecyl sulfate polyacrylamide gel electropheresis analyzer (SDS-PAGE; ATTO, Tokyo, Japan) and transferred to 0.45-μm polyvinylidene fluoride (PVDF) films through the TE-22 protein transfer printer (GE; Cytiva, New York, NY, USA). The mixtures were incubated with the primary antibodies (Occludin, Claudin-1, ZO-1, 1:2000, Beyotime, Shanghai, China) overnight at 4 °C after being sealed for 1 h in 5% skim milk made with TBST buffer. Horseradish peroxidase (HRP)-conjugated secondary antibodies (1:2000, Beyotime) were then added and incubated for 1 h on a shaking table. The samples were imaged with an ECL kit (Beyotime, Shanghai, China), and the bands were visualized by Image J software (Oberkochen, Germany). Table 1 contains a list of the primer sequences that were investigated. 

### 2.11. Effect of Planktonic and Biofilm L. plantarum Y42 on L. monocytogenes ATCC 19115

#### 2.11.1. The Growth and Clearance of *L. monocytogenes* ATCC 19115 Biofilms

We conducted the following tests to assess the inhibitory effects of planktonic and biofilm *L. plantarum* Y42 on the growth and clearance of *L. monocytogenes* ATCC 19115 biofilms. Planktonic or biofilm *L*. *plantarum* Y42 and *L. monocytogenes* ATCC 19115, at an initial concentration of 2.0 × 10^9^ CFU/mL, were inoculated in sterile TSB medium (1: 100, *v*/*v*), then transferred 1 mL to 12-well polystyrene microtiter plates and incubated at 37 °C for 24 h. In the experiment of clearing *L. monocytogenes* ATCC 19115 biofilms, 1 mL TSB medium inoculated with *L. monocytogenes* ATCC 19115 (1: 100, *v*/*v*) was added to 12-well polystyrene microtiter plates and incubated at 37 °C for 24 h to form biofilms first. Then 900 μL of sterile TSB medium and 100 μL 1.0 × 10^9^ CFU/mL of planktonic or biofilm *L*. *plantarum* Y42 were resuspended in sterile TSB medium and incubated at 37 °C for 12 h. *L. monocytogenes* ATCC 19115 without *L. plantarum* Y42 was used as a control. After culture, the broth was discarded and the biofilms were washed with PBS (pH 7.3) three times, *L. monocytogenes* ATCC 19115 was counted with PALCAM medium (Solarbio, Beijing, China) by using the viable plate count method.

#### 2.11.2. Adhesion of *L. monocytogenes* ATCC 19115 on HT-29 Cells

The planktonic/biofilm *L*. *plantarum* Y42 and *L. monocytogenes* ATCC 19115 were resuspended in RPMI 1640 medium (without antibiotics or fetal bovine serum) to a final concentration of 2.0 × 10^9^ CFU/mL. Added 2 × 10^5^ cells/mL of HT-29 cells to the 12-well polystyrene microtiter plate and cultured at 37 °C in a humidified atmosphere containing 5% CO_2_ and 95% air until a cell monolayer is formed. The broth was discarded, and the cells were gently rinsed with PBS (pH 7.3) three times. A 100 μL suspension sample of planktonic or biofilm *L*. *plantarum* Y42 and *L. monocytogenes* ATCC 19115 were added to the wells, respectively. The RPMI 1640 medium was refilled to 1 mL and incubated for 2 h. The broth was discarded, and the cells were washed with PBS (pH 7.3) to remove the unadhered bacteria, 500 μL 0.5% TritonX-100 was added, and the cells were evenly blown to from lysate. The solution was transferred to 4.5 mL of normal saline for gradient dilution. In addition, the cells were treated with antibiotics before the addition of TritonX-100 to assess the invasion ability of *L. monocytogenes* ATCC 19115 on HT-29 cells. The number of *L. monocytogenes* ATCC 19115 adhering to and invading HT-29 cells was counted with PALCAM medium by using the viable plate count method. Inflammatory factors and tight junction proteins were determined by the method described in Section “Secretion of inflammatory factors.”

### 2.12. Statistical Analysis

IBM SPSS Statistics 20.0.0 (Chicago, IL; USA) was used to conduct an analysis of variance (ANOVA) at a 95% confidence level, while GraphPad Prism 8 (San Diego, CA, USA) was used to visualize the results. The results of each experiment were given as a mean ± standard deviation after being repeated three times. *p < 0.05; ** *p* < 0.01; *** *p* < 0.001; ns is not significant. Columns labeled with different lowercase letters are significantly different at *p* < 0.05.

## 3. Results

### 3.1. Influence Factors on the Biofilm Formation of L. plantarum Y42 

**Figure 1:**
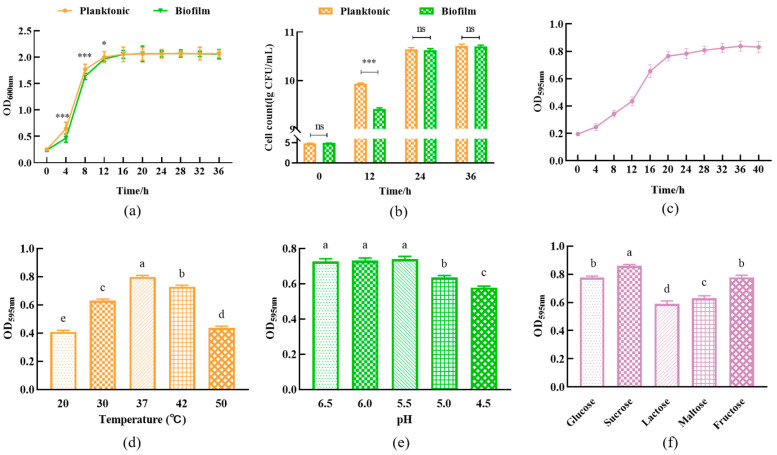
Growth curves (**a**) and viable counts (**b**) of *L. plantarum* Y42 in the planktonic and biofilm forms. Biofilm formation of *L. plantarum* Y42 (**c**). Effects of different temperatures (**d**), pH (**e**), and carbon sources (**f**) on the formation of *L. plantarum* Y42 biofilm. * *p* < 0.05; *** *p* < 0.001; ns is not significant. Columns labeled with different lowercase letters are significantly different at *p* < 0.05.

The planktonic *L. plantarum* Y42 at the log phase (0–12 h) grew faster than the biofilm strains (Figure 1a), suggesting that *L. plantarum* Y42 has access to more nutrients and grows rapidly during shaken culture. And there was no significant difference (*p* > 0.05) in the quantity of both planktonic and biofilm *L. plantarum* Y42 at the same incubation time after they entered the stationary phase (Figure 1b). As shown in Figure 1c, the quantity of *L. plantarum* Y42 biofilm formation increased with the increase in culture time and reached its maximum at 36 h. Then we determined the factors affecting the formation of *L. plantarum* Y42 biofilm, and the results showed that the biofilm formation of *L. plantarum* Y42 was affected by the initial pH of the culture medium, the carbon source, and the culture temperature (Figure 1d–f). The quantity of *L. plantarum* Y42 biofilm maximized at 37 °C. When the initial pH value of the medium was below 5, the quantity of biofilms significantly decreased (*p* < 0.05). And sucrose as a carbon source significantly promoted the biofilm formation of *L. plantarum* Y42. These results are consistent with previous studies [24,25,26]. 

### 3.2. Morphological Observations of L. plantarum Y42 in Biofilm and Planktonic Forms

**Figure 2:**
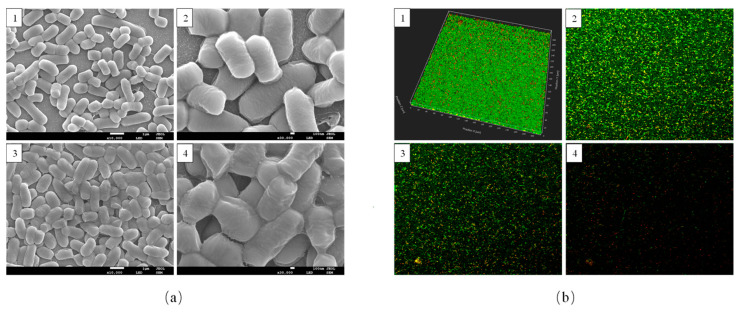
Scanning electron microscopy (SEM) images (**a**) of *L. plantarum* Y42 in the planktonic (**a1**, **a2**) and biofilm (**a3**,**a4**) forms. The images were taken at 10,000× (**a1**,**a3**) and 30,000× (**a2**,**a4**) magnification, respectively. CLSM images of *L. plantarum* Y42 biofilms (**b**). 3D images of biofilm stained by SYTO 9 (live cells, green) and PI (dead cells, red) (**b1**). The distribution of live and dead cells at different heights, where (**b2**–**b4**) were the surface, middle, and bottom positions, respectively.

The morphologies of planktonic and biofilm *L. plantarum* Y42 were observed under SEM. The *L. plantarum* Y42 planktonic cells mostly existed independently as individual or paired bacteria, and the cell surface was smooth without excessive adhesive substances (Figure 2(a1,a2)). The *L. plantarum* Y42 biofilm cells in Figure 2(a3,a4) were clearly wrapped by a thick layer of extracellular polymer matrices and had a rough surface without evident outlines. The cells interconnected via the surface adherent substances to form a three-dimensional network structure, which indicates the evident characteristics of biofilms. It was reported that *Streptococcus mutans* [27] and *Tetragenococcus halophilus* [17] in biofilm form have similar structures. 

CLSM can provide information about cell localization in biofilms [27]. The 3D graph in Figure 2(b1) showed that *L. plantarum* Y42 formed a dense layer of biofilms with a thickness of ~18 μm on the glass slides after 36 h of culture. And the 2D graph in Figure 2(b2–b4)) showed that from the surface to the bottom of the biofilms of *L. plantarum* Y42, the number of living cells gradually decreases while the number of dead cells gradually increases. This may be because cells near the surface have access to more nutrients and multiply faster, while the lack of nutrients at the bottom of the bacterial biofilm and the accumulation of harmful metabolites such as acids are detrimental to cell survival [28]. Moreover, the CLSM image at the bottom of the biofilms showed low fluorescence intensity, which may be because the compaction of the biofilms led to non-uniform staining [17].

### 3.3. Surface Properties of L. plantarum Y42 in Biofilm and Planktonic Forms

**Figure 3:**
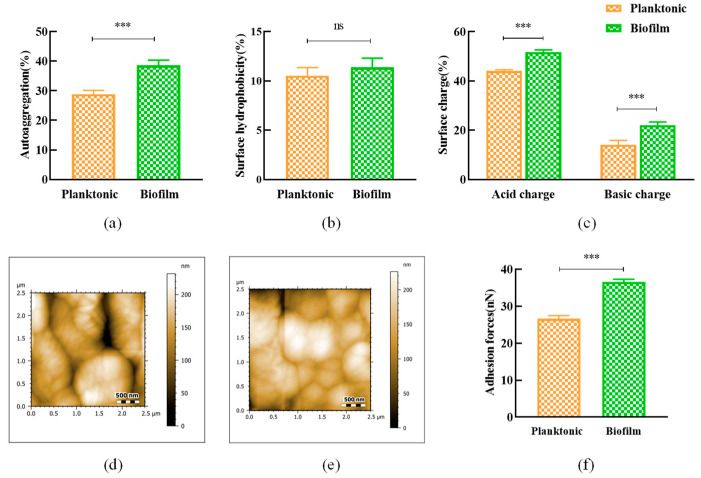
Cell surface properties. Automatic aggregation capability (**a**), hydrophobicity (**b**) and surface charges (**c**) of *L. plantarum* Y42 in the planktonic and biofilm forms. AFM images of *L. plantarum* Y42 in the planktonic (**d**) and biofilm (**e**) forms. Adhesion force on the surface of *L. plantarum* Y42 in the planktonic and biofilm forms (**f**). *** *p* < 0.001; ns is not significant.

As shown in Figure 3a–c, both the automatic aggregation capability and surface acid-base charge of *L. plantarum* Y42 in the biofilm form were significantly higher than those in the planktonic form (*p* < 0.001). The AFM result showed that the biofilm cells had a rough surface compared with the planktonic cells (Figure 3d,e). And the surface adhering ability of *L. plantarum* Y42 in the biofilm form was significantly stronger than that in the planktonic form by analyzing the force curves (*p* > 0.05; Figure 3f). These surface properties of bacteria were closely related to the abilities of adhesion and aggregation [29].

### 3.4. Tolerance of L. plantarum Y42 in Biofilm and Planktonic Forms to Harsh Environmental Conditions

**Figure 4:**
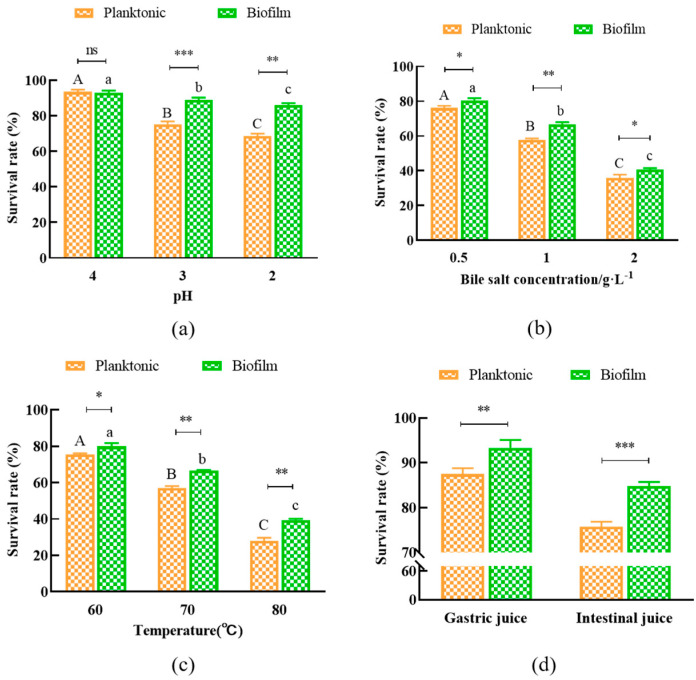
Effects of pH (**a**), bile salt concentration (**b**), temperature (**c**), simulated artificial gastric juice, and intestinal fluid (**d**) on the growth of planktonic and biofilm *L. plantarum* Y42. * *p* < 0.05; ** *p* < 0.01; *** *p* < 0.001; ns is not significant. Columns labeled with different lowercase letters are significantly different at *p* < 0.05, where A, B, C represent the planktonic group, a, b, c represent the biofilm group.

The stress tolerances to acids, bile salts, a range of temperatures, and simulated artificial gastro-intestinal fluids of planktonic and biofilm *L. plantarum* Y42 were measured. The survival rate of *L. plantarum* Y42 in the biofilm form was higher than that in the planktonic form when the pH values of the culture condition ranged from 3 to 2 (Figure 4a). Although high bile salt concentration and temperature decreased the survival rate of *L. plantarum* Y42 in both forms, biofilm *L. plantarum* Y42 exhibited a higher survival rate under the same conditions compared to that in the planktonic form, as shown in Figure 4b,c. Additionally, the survival rate of *L. plantarum* Y42 in the biofilm form was higher than that in the planktonic form when treating with the simulated gastric fluid (93.32% vs. 87.47%) and intestinal fluid (84.82% vs. 75.73%) separately (Figure 4d). So, *L. plantarum* Y42 in biofilm form exhibited higher tolerance to harsh environmental conditions, which was consistent with previous research [30,31].

### 3.5. Vacuum Lyophilization of L. plantarum Y42 in Biofilm or Planktonic Forms

**Figure 5:**
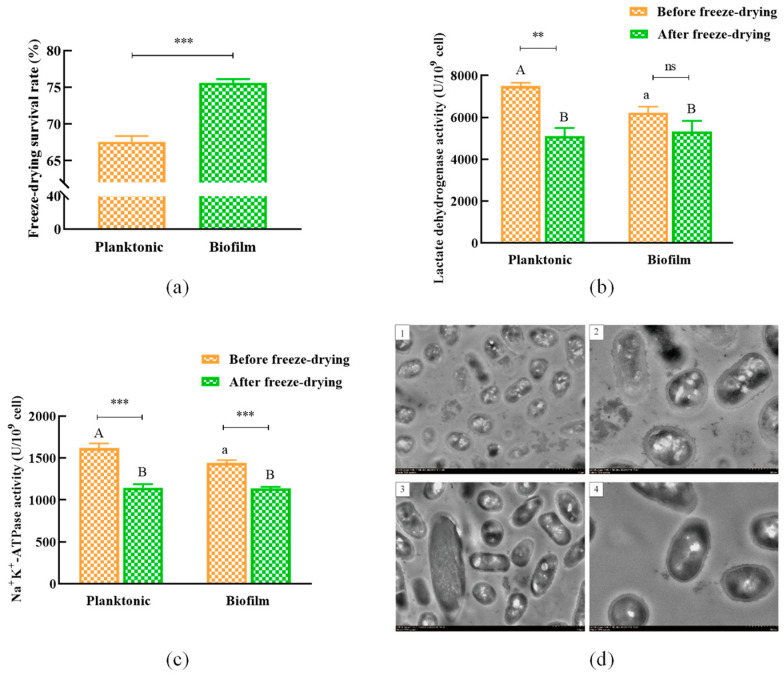
Effects of vacuum lyophilization on the viability of planktonic and biofilm *L. plantarum* Y42. Lyophilized survival rate (**a**), LDH (**b**), and Na^+^ K^+^-ATP (**c**) enzyme activities before and after lyophilization. TEM images of *L. plantarum* Y42 in the planktonic (**d1**,**d2**) and biofilm (**d3**,**d4**) forms after vacuum lyophilization (**d**). The images were taken at 1000× (**d1**,**d3**) and 2000× (**d2**,**d4**) magnification, respectively. ** *p* < 0.01; *** *p* < 0.001; ns is not significant. Columns labeled with different lowercase letters are significantly different at *p* < 0.05, where A, B represent the group of before freeze-drying, a represent the group of after freeze-drying.

The survival rates of *L. plantarum* Y42 in planktonic and biofilm forms were 67.58% and 75.60%, respectively, after vacuum lyophilizing (Figure 5a), with a significant difference (*p* < 0.01). The lactate dehydrogenase and Na^+^ K^+^-ATPase activities dropped by 31.92% and 29.16%, respectively, in the planktonic form after the vacuum lyophilization, while they dropped by only 14.65% and 21.03%, respectively, in the biofilm form (Figure 5b,c). Moreover, the TEM result showed that the cell membrane of *L. plantarum* Y42 in the planktonic form was destroyed after vacuum lyophilization (Figure 5(d1,d2)), while the bacterial cells in the biofilm form basically reserved the intact rod shape after lyophilization (Figure 5(d3,d4)). This may be attributed to the protective effect of the extracellular polymeric matrix on the surface of biofilm bacterial cells [32].

### 3.6. Expressions of Inflammatory Factors and Tight Junction Proteins 

**Figure 6:**
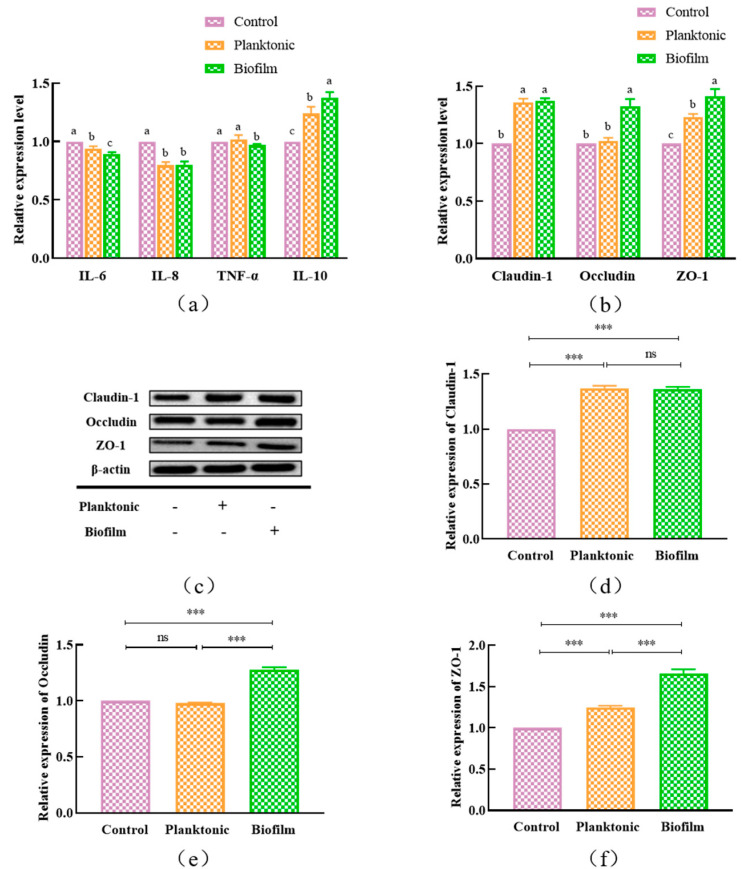
The expressions of cytokines related to inflammation (**a**) and the expressions of tight junction proteins (**b**) in HT-29 cells treated with planktonic and biofilm *L. plantarum* Y42 by using RT-qPCR and WB (**c**–**f**). *** *p* < 0.001; ns is not significant. Columns labeled with different lowercase letters are significantly different at *p* < 0.05.

It was reported that most probiotics can play a protective role in maintaining intestinal tract barrier functions by adjusting the expressions of inflammatory factors and intercellular tight junction proteins, and adhesion is the key step for *L. plantarum* to play the probiotic role in vivo [9,33]. In our results, the adhesion rate of *L. plantarum* Y42 to HT-29 cell monolayers was significantly higher in the biofilm form than that in the planktonic form (92.09% vs. 85.60%, *p* < 0.05) (Figure S1). The HT-29 cells treated with planktonic or biofilm *L. plantarum* Y42, respectively, exhibited lower RNA expression of IL-6, IL-8, and TNF-α compared to the control group, and the RNA expression of IL-6 and TNF-α of HT-29 cells was lower in the biofilm group than that in the planktonic group under the same condition. On the contrary, the RNA expression of IL-10 in HT-29 cells treated with either planktonic or biofilm *L. plantarum* Y42 was significantly increased, and it had higher expression in the biofilm group (Figure 6a). Moreover, the treatment with *L. plantarum* Y42 in two forms promoted the expression of the three tight junction proteins in HT-29 cells. Specifically, the expressions of Occludin and ZO-1 were significantly higher in the biofilm group. And the detection results were consistent between the two methods of RT-qPCR (Figure 6b) and western blot (WB) (Figure 6c–f). These results indicate the biofilm *L. plantarum* Y42 outperforms the planktonic state in protecting intestinal barrier functions. 

### 3.7. Effect of Planktonic and Biofilm L. plantarum Y42 on L. monocytogenes ATCC 19115

#### 3.7.1. The Growth and Clearance of *L. monocytogenes* ATCC 19115 Biofilms

**Figure 7:**
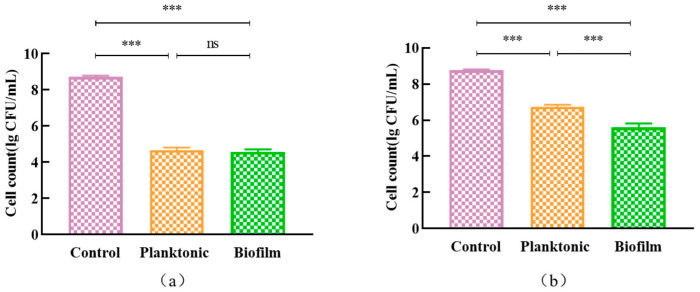
The inhibitory effects of planktonic and biofilm *L. plantarum* Y42 on the growth (**a**) and clearance (**b**) of *L. monocytogenes* ATCC 19115 biofilms. *** *p* < 0.001; ns is not significant.

We validated whether the *L. plantarum* Y42 in the two forms was different in inhibiting the growth of *L. monocytogenes* ATCC 19115. Although there was no significant difference in the inhibition of *L. monocytogenes* ATCC 19115 biofilm formation after coculture with planktonic or biofilm *L. plantarum* Y42 (Figure 7a), the biofilm *L. plantarum* Y42 had a stronger scavenging ability on the *L. monocytogenes* ATCC 19115 biofilm compared to the planktonic *L. plantarum* Y42 (*p* < 0.001) (Figure 7b). This indicates that planktonic and biofilm *L. plantarum* Y42 have an inhibitory effect on the growth and biofilm formation of *L. monocytogenes* ATCC 19115.

#### 3.7.2. The Inhibitory Effects of Planktonic and Biofilm *L. plantarum* Y42 on the Adhesion and Invasion of *L. monocytogenes* ATCC 19115 of HT-29 Cell Monolayers

**Figure 8:**
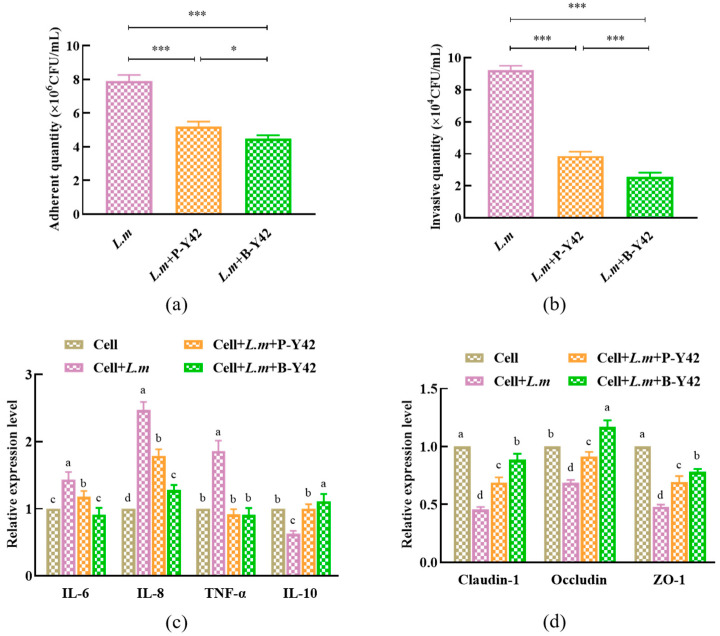
Effects of planktonic and biofilm *L. plantarum* Y42 on the adhesion (**a**) and invasion (**b**) of *L. monocytogenes* ATCC 19115 to HT-29 monolayer cells. The expressions of cytokines related to inflammation (**c**) and tight junction proteins (**d**) in HT-29 cells by using RT-qPCR. * *p* < 0.05; *** *p* < 0.001; ns is not significant. Columns labeled with different lowercase letters are significantly different at *p* < 0.05.

Adhesion is the first step in pathogenic bacterial invasion of host cells (i.e., intestinal epithelial cells) [34]. In this study, the adhesion number of *L. monocytogenes* ATCC 19115 co-cultured with planktonic and biofilm *L. plantarum* Y42, respectively, on a HT-29 cell monolayer was significantly lower than that of *L. monocytogenes* ATCC 19115 alone on the same monolayer (Figure 8a), and the same was true for the number of *L. monocytogenes* ATCC 19115 invading HT-29 cells (Figure 8b). The adhesion and invasion abilities of *L. monocytogenes* ATCC 19115 to HT-29 cell monolayers were interrupted by either planktonic or biofilm *L. plantarum* Y42, and *L. plantarum* Y42 in the biofilm form showed a higher inhibiting effect on *L. monocytogenes* ATCC 19115 than in the planktonic form. 

As for the inflammatory cytokine expression, the HT-29 cells treated by *L. monocytogenes* ATCC 19115 combined with planktonic or biofilm *L. plantarum* Y42, respectively, showed lower RNA expression of IL-6, IL-8, and TNF-α, compared to those of HT-29 cells treated only by *L. monocytogenes* ATCC 19115 (Figure 8c), and the expressions of IL-6 and IL-8 in the biofilm group were considerably reduced compared to the planktonic group (*p* < 0.05). However, the RNA expression of IL-10 was contrary to that of IL-6 and IL-8. Furthermore, the addition of planktonic and biofilm *L. plantarum* Y42 relieved the downregulation of tight junction proteins (Claudin-1, Occludin, and ZO-1) caused by *L. monocytogenes* ATCC 19115, and the biofilm *L. plantarum* Y42 was more effective than the planktonic state (Figure 8d), especially the biofilm group’s Occludin expression, which was substantially higher than the control group (*p* < 0.05). So, the biofilm *L. plantarum* Y42 is more effective in relieving the inflammatory reaction and intestinal barrier dysfunction of HT-29 cell monolayers induced by *L. monocytogenes* ATCC 19115. 

## 4. Discussion

Most bacteria naturally grow in biofilms, which make them more resistant to medications, harsh conditions, and the host’s immune defense mechanisms [30]. Bacterial biofilms are affected by environmental conditions and bacterial gene expressions, and their formation is regulated by different pathways and mechanisms [35]. Lactic acid bacteria have the capacity to form biofilms, and this capacity is largely strain-specific [2]. 

In this study, we found that some incubating conditions, including time, temperature, pH, and the carbon source of the culture medium, all affected the biofilm formation of *L. plantarum* Y42. Yao et al. [17] reported similar outcomes as well. Moreover, there was a significant correlation between the cell density and biomass of *L. plantarum* Y42 biofilms. A slight increase in biofilms was observed when the cell density reached stability, which may be due to the accumulation of the exo-polymatrix, caused by cell secretion and death [36]. When the pH of the medium dropped below 5, the quantity of biofilms considerably decreased (*p* < 0.05), demonstrating that an acid environment can effectively prevent *L. plantarum* Y42 from forming biofilms. Acid stress can impact the aggregation of biofilms by reducing the levels of exopolymers, such as proteins and polysaccharides, in the biofilm matrix. It can also change the matrix’s charge and morphological properties [37,38]. Carbohydrates in the culture medium have been reported to regulate the biosynthetic pathway of *Lactobacillus* exopolysaccharides [39], it has been demonstrated that sucrose alters the polysaccharide content in the biofilm matrix, facilitating bacterial adhesion and biofilm development [25], and fructose as the carbon source improved the biofilm quantity and total surface antigenicity of *Lacticaseibacillus rhamnosus* [40]. In this study, sucrose and fructose exhibited promoting effects on the biofilm formation of *L. plantarum* Y42.

The bacteria embodied in the biofilm can be tolerant to adverse environmental conditions [41]. Our study showed that *L. plantarum* Y42 in biofilm form, compared with that in planktonic form, was more tolerant to acids, bile salts, temperature, and gastro-intestinal fluids. It was reported that some microorganisms will quickly form biofilms to protect cells when exposed to adverse environments [42]. *Tetragenococcus halophilus* was found to survive better in the biofilm form than in the planktonic form when subjected to heat and oxidative stress, according to Yao et al. [17]. One of the elements contributing to the high rate of biofilm’s survival in unfavorable environments may be the protective effect of the extracellular polymeric matrix covering the biofilm’s surface. The biofilm of *L. plantarum* Y42 had a rough surface and blurry morphology due to a thick layer of extracellular polymer matrices.

Vacuum lyophilization is the most practical and efficient technology so far for the preservation of probiotic strains, but it will cause some mechanical damages to the bacterial cell membrane, decrease the survival rate of lyophilized bacteria, and inactivate some key enzymes, such as lactate dehydrogenase and Na^+^ K^+^-ATPase [43,44]. In this study, the survival rate of lyophilized *L. plantarum* Y42 in the biofilm form was higher than that in the planktonic form, and the decreasing rates of enzymatic activities after lyophilization in the planktonic *L. plantarum* Y42 were far higher compared with the biofilm cells. Interestingly, the activities of the lactate dehydrogenase and Na^+^ K^+^-ATPase of planktonic *L. plantarum* Y42 were higher than those of the biofilm state before lyophilization; this may be due to the fact that the activities of bacterial cells in the biofilm were restricted by nutrition lack, harmful metabolite accumulation, and quorum sensing [45]. After vacuum lyophilization, *L. plantarum* Y42’s planktonic cell membrane was damaged, and a large quantity of intracellular materials flowed out, which severely affected the permeability of cell membranes. In comparison, the cells in the biofilm reserved the intact rod shape after lyophilization, with only a small number of cells rupturing. This was consistent with the previous result that the activity of Na^+^ K^+^-ATPase decreased at a higher rate in the planktonic *L. plantarum* Y42. These results suggested that biofilm formation can effectively protect strains. According to Jingjing et al. [4], encouraging *L. plantarum* LIP-1 biofilm development may result in greater lyophilized survival rates. Bacterial biofilm formation successfully prevented the entry of macromolecular compounds and raised the diffusion resistance of small molecules to biofilm while also enhancing bacterial cell-to-cell communication and minimizing environmental damage [46]. 

The capacity of bacteria to adhere is directly tied to biofilm formation; probiotics in biofilm form are more conducive to colonizing the intestinal mucosa. [47]. The micropopulations colonizing the gastrointestinal tract can regulate the expression of some inflammatory factors and tight junction proteins and thereby play a critical role in maintaining intestinal barrier functions and the host’s health [48]. In this study, the biofilm *L. plantarum* Y42 exhibited stronger adhesion to HT-29 cell monolayers compared to planktonic strains, which was confirmed by the AFM results. Liu et al. [34] reported that *Lactiplantibacillus plantarum* L-ZS9 in the biofilm form exhibited higher transcription levels of adhesion-related genes than those in the planktonic form; this was in line with the fact that planktonic strains were less likely to adhere to HT-29 cells than the biofilm *L. plantarum* L-ZS9, which had a 1.5 times greater adhesion rate. In this study, biofilm *L. plantarum* Y42 promoted intestinal barrier functions by enhancing the expression of tight junction proteins. The extracellular polymeric substances during the biofilm formation of *L. plantarum* Y42 improved the self-aggregating ability, surface hydrophobicity, and surface acid/base charge, which were closely related to the abilities of adhesion and aggregation and had a significant impact on the initial adhesion [49]. And these extracellular polymer matrices can specifically bind with the pattern recognition receptors (PRR) of the intestinal epithelial cells [50] and regulate nuclear factor-kappa B (NF-κB), mitogen activation protein kinase (MAPK), and peroxisome proliferators-activated receptor γ (PPARγ) signaling pathways [51], thereby relieving inflammation and enhancing intestinal barrier function [52]. 

The intestinal mucosal barrier can prevent pathogenic microorganisms from entering the host via the intestinal mucosa, and thus stop systemic inflammation and immune responses [53]. It was reported that once the biofilm formed, probiotics exhibited an enhanced inhibiting effect on pathogens [54]. In this study, the biofilm *L. plantarum* Y42 showed a better inhibiting effect on the adhesion and invasion of *L. monocytogenes* ATCC 19115 to HT-29 cell monolayers. Bujnakova et al. [55] found that *Lactobacillus rhamnosus* 183 can strongly form biofilms on the surface of polystyrene and can inhibit *Escherichia coli*, *Salmonella,* and other pathogens after biofilm formation. This may be related to the protective effect of the biofilm extracellular polymeric matrix. The biofilm *L. plantarum* Y42 was more effective in relieving the inflammatory reactions and barrier function injuries of HT-29 cells caused by *L. monocytogenes* ATCC 19115. According to Chervinets et al. [56], the development of biofilm can facilitate probiotics colonizing the intestinal epithelium, prolong the retention time, and improve the defense ability of the host, thereby preventing the invasion of pathogens and enhancing the intestinal barrier function [57,58]. The biofilm *L. plantarum* Y42 expressed more extracellular polysaccharides, proteins, and other polymeric substances, which may contribute to the decreased expression of toxic proteins and invasive ability of *L. monocytogenes* ATCC 19115, just like the report by Lebeer et al. [59]. On the other hand, the extracellular polymers could do favor to the competition of *L. plantarum* Y42 with *L. monocytogenes* ATCC 19115 for adhesion sites on the surface of intestinal cells so as to protect intestinal barrier functions [60].

## 5. Conclusions

*L. plantarum* Y42 showed stronger probiotic functions after biofilm formation, including environmental tolerance, the lyophilized survival rate, adhering ability to HT-29 cells, enhancing the intestinal barrier, and an inhibitory effect on *L. monocytogenes* ATCC 19115. The more abundant extracellular polymeric substances produced by *L. plantarum* Y42 during its biofilm formation wrapped the bacterial cells and played a certain protective role, thus enhancing the probiotic functions of the strain. In addition to the extracellular polymeric substances, whether *L. plantarum* Y42 produces other different metabolites during biofilm formation will be further studied. The probiotic function of probiotics is associated with their effector molecule. So, exploring the difference in metabolites between the biofilm and planktonic forms of *L. plantarum* Y42 is important for the effective application of the strain. In general, this study will theoretically underline the exploitation and utilization of biofilm-state probiotics. 

## Figures and Tables

**Table 1:** Primers used in real-time quantitative PCR.

Gene	Primer Sequence (5′-3′)
GAPDH	F: GGAAGGTGAAGGTCGGAGTC
	R: TCAGCCTTGACGGTGCCATG
IL-6	F: AAGCCAGAGCTGTGCAGATGAGTA
	R: TGTCCTGCAGCCACTGGTTC
IL-8	F: ACACAGAGCTGCAGAAATCAGG
	R: GGCACAAACTTTCAGAGACAG
TNF-α	F: CTGCCTGCTGCACTTTGGAG
	R: ACATGGGCTACAGGCTTGTCACT
IL-10	F: GAGATGCCTTCAGCAGAGTGAAGA
	R: AGTTCACATGCGCCTTGATGTC
β-actin	F: ACTCTGGTGATGGTGTTAC
	R: GGCTGTGATCTCCTTCTG
Claudin-1	F: CATACTCCTGGGTCTGGTTGGT
	R: GACAGCCATCCGCATCTTCT
Occludin	F: ACGGCAGCACCTACCTCAA
	R: GGGCGAAGAAGCAGATGAG
ZO-1	F: CTTCAGGTGTTTCTCTTCCTCCTC
	R: CTGTGGTTTCATGGCTGGATC

## Data Availability

All related data and methods are presented in this paper. Additional inquiries should be addressed to the corresponding author.

## Supplementary Materials

The following supporting information can be downloaded at: https://www.mdpi.com/article/10.3390/foods12071516/s1, Figure S1: The adhesion rates of *L. plantarum* Y42 in the planktonic and biofilm forms to HT-29 cells. **p* < 0.05; ** *p* < 0.01; *** *p* < 0.001; ns is not significant.
