# Supplementary material for: Phenotypic Traits and Probiotic Functions of Lactiplantibacillus plantarum Y42 in Planktonic and Biofilm Forms
Source: Foods. 2023 Apr 3;12(7):1516. doi: 10.3390/foods12071516 (PMC10093976; doi:10.3390/foods12071516)
Supplement: Supplementary file 1 [file foods-12-01516-s001.zip › foods-2291978-supplementary.pdf]

foods

Supplementary Materials

Phenotypic Traits and Probiotic Functions of *Lactiplantibacillus plantarum* Y42 in Planktonic and Biofilm Forms

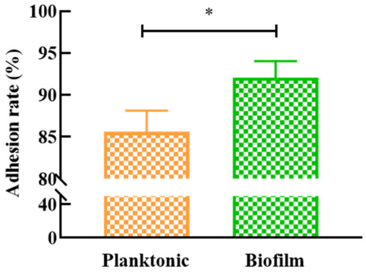

Figure S1. The adhesion rates of *L. plantarum* Y42 in the planktonic and biofilm forms to HT-29 cells. *$p < 0.05$; ** $p < 0.01$; *** $p < 0.001$; ns is not significant.
